# Editorial: Challenges in implementing digital health in public health settings in low and middle income countries

**DOI:** 10.3389/fpubh.2022.1090303

**Published:** 2023-01-10

**Authors:** Mona Duggal, Alison El Ayadi, Bhanu Duggal, Nancy Reynolds, Covadonga Bascaran

**Affiliations:** ^1^Advanced Eye Centre, Post Graduate Institute of Medical Education and Research (PGIMER), Chandigarh, India; ^2^Department of Obstetrics, Gynecology and Reproductive Sciences, University of California, San Francisco, San Francisco, CA, United States; ^3^Department of Cardiology, OSD, NITI Aayog, All India Institute of Medical Sciences, Rishikesh, India; ^4^School of Nursing, Johns Hopkins University, Baltimore, MD, United States; ^5^International Centre for Eye Health, London School of Hygiene and Tropical Medicine, University of London, London, United Kingdom

**Keywords:** digital health, COVID-19, challenge, mHealth, healthcare

Healthcare challenges in low- and middle-income (LMICs) have been the focus of many digital initiatives that have aimed to ensure consistent implementation of these services. During the COVID-19 pandemic, several lockdowns were imposed globally by government authorities to contain the spread of the virus. This triggered a rapid effort to integrate digital technologies into the existing health systems of LMICs (1). Digital services have the potential to improve access and care coordination across health facilities by overcoming the conventional obstacles and weaknesses of traditional systems. To promote better adoption of digital health tools the challenges need to be understood and strategies to overcome barriers must be evaluated. Hence the aim of this Research Topic was to identify specific organizational and related barriers in implementing digital health in public health settings in LMICs and further explore facilitators for successful implementation of digital technologies.

During the pandemic, many countries have been developing mHealth apps to identify prevalent symptoms, self-assessment, contact tracing and disseminating information. This helped to minimize exposure avoiding physical interaction between patients and health workers. Sujarwoto et al., systematically reviewed COVID-19 related mHealth apps in Indonesia and found the main uses were disseminating information, self-risk assessment, providing an online community forum and teleconsultation. They highlighted the challenges related to data security, privacy, integration and infrastructure. Tjiptoatmadja and Alfian, assessed awareness, perception, and willingness to use telepharmacy services and reported that over half of the study participants, had heard about telepharmacy and the majority of them had a positive perception and were willing to use telepharmacy services.

Lee et al. focused upon developing sustainable genomics surveillance programs in LMICs through adoption of a target operating model in a stepwise manner. The authors discussed the various barriers faced by such programs e.g., resource limitations, workforce strain, unreliable supply chains and lack of enduring champions, which exacerbate implementation and sustainability challenges. In other work, Iyamu et al. (2), discussed various technical (fragmented and unsustainable systems, lack of clear standards, and unreliability of available data, infrastructure gaps, and workforce capacity gaps) and non-technical challenges (ethics, policy and governance, health equity, resource gaps, and quality of evidence) in the development of digital public health interventions.

One of the challenges of implementation of digital health in LMICs is cost. A detailed protocol on determining the costs of a large mHealth job aid for health and nutrition in India is discussed by Shukla and Kapur, through a behavior change communication tool known as ICDS-CAS (Integrated Child Development Services-Common Application Software). This research used the Activity Based Costing—Ingredients (ABC-I) method approach with aims to break down the program into a sum of mutually exclusive and exhaustive activities. A brief review by Yogesh and Karthikeyan, discussed the future trends and directions in health informatics, noting that there are no proven design blueprints for a comprehensive infrastructure. The authors also report that big data is playing an important role in health informatics, where a large amount of data related to healthcare is generated.

A report of two case studies from India by Senjam and Primo, focused upon the challenges and enablers for smartphone use by persons with vision loss during the COVID-19 pandemic. The most important enabling factors found were the presence of a screen reader, data connection of the mobile and the ability to assess multiple languages. Conversely, frequent challenges included poor battery backup, frequent unwanted ads or pop-ups unreadable by a screen reader, and slow or unresponsive screen readers. In a study from Nepal, Sankhi et al. (3) interviewed blind teenagers about the challenges experienced when using smartphones as assistive devices. Lack of training in using the devices was an issue and screen-readers' limitations in correctly pronouncing the local language are highlighted.

In another study on eye health in Pakistan Khan A. A. et al., had the goal of increasing eye health program coverage and effectiveness by using various strategies including digital data monitoring and visualization. The authors show that modifications of the program based on ongoing review of data and evidence can improve the program, specifically attendance to hospital appointments. The continued monitoring of gender imbalances in program data is another advantage of the system. Burton et al. (4), Mercer et al. (5), and Ramke et al. (6), argue that in order to tackle Sustainable Development Goal (SDG) 5—Gender Equality—it is important to monitor how a program is reaching each gender, and take necessary measures to make it easier for all patients to access assessment and treatment.

In a review of literature on AI approaches for promoting maternal and neonatal health in low resource settings by, Khan M. et al., pointed out to unreliable data collection and explainability in AI is a major roadblock to its widespread adoption of, of AI/ML algorithms,.

Another research article by Chai et al., demonstrated the use of a 5G-based robot-assisted remote ultrasound system (MGIUS-R3; Wisonic Medical Technology Co., Ltd., Shenzhen, China) that was used for the tele-examination for patients with disabilities at a remote care center. The same patients were examined by two independent sonographers using 5G-based robot-assisted remote ultrasound. The authors concluded that the use of a 5G-based robot-assisted remote ultrasound system is feasible in patients with disabilities at a remote care center and results in similar diagnostic efficacy to traditional bedside ultrasound, the gold standard. A similar article by Lim et al., evaluated the reliability and accuracy of 2D photogrammetry, as compared to direct measurement (gold standard). The authors discuss that three facial dimensions cannot be measured reliably and accurately using the 2D photogrammetry method because of poor inter-rater reliability of 2D photogrammetry.

In another study on remote monitoring Jain et al., reported the success of monitoring and holistic care of healthcare workers affected with mild COVID-19 and residing under home isolation through the use of digital technology. Healthcare workers faced additional challenges when compared to the general population besides a greater risk of becoming infected, harboring a higher virological burden, and a potentially more serious illness, they have additional work-related and psychosocial stressors.

Taken together, this collection of articles highlight the need to test the validity and reliability of digital health tools to streamline their function and design them according to the needs of programs in low income countries in order to minimize implementation challenges. Policymakers must consider usefulness, usability, integration, and infrastructure issues to improve their digital health functions. For full-scale sustainability, financing for all aspects of digital health solutions needs to be integrated into routine health budgets and the budgeting processes.

## Author contributions

MD wrote the editorial. All authors co-edited the Research Topic, contributed to the editorial editing, and approved the submitted version.
